# A pandemic treaty, revised international health regulations, or both?

**DOI:** 10.1186/s12992-021-00779-0

**Published:** 2021-11-06

**Authors:** Ronald Labonté, Mary Wiktorowicz, Corinne Packer, Arne Ruckert, Kumanan Wilson, Sam Halabi

**Affiliations:** 1 School of Epidemiology and Public Health, University of Ottawa, 600 Peter Morand Crescent, Ottawa, ON K1G 5Z3 Canada; 2 Dahdaleh Insitute for Global Health Research, York University, 88 The Pond Road, Suite 2150, Toronto, ON M3J 2S5 Canada; 3 School of Health Policy and Management, York University, 4700 Keele St, Toronto, ON M3J 1P3 Canada; 4 Department of Medicine, University of Ottawa, Bruyère Research Institute and Ottawa Hospital Research Institute, Civic Campus, 1053 Carling Avenue, Box 684, Administrative Services Building, Ottawa, ON K1Y 4E9 Canada; 5 O’Neill Institute for National and Global Health Law, Georgetown University, 600 New Jersey Avenue, NW, Washington, DC, 20001 USA; 6 Colorado School of Public Health at Colorado State University, Sage Hall, 700 South Mason St., Fort Collins, CO 80523 USA

**Keywords:** Pandemic treaty, International health regulations (IHR), COVID-19, Vaccine, Equity

## Abstract

**Background:**

A special session of the World Health Assembly (WHA) will be convened in late 2021 to consider developing a WHO convention, agreement or other international instrument on pandemic preparedness and response – a so-called ‘Pandemic Treaty’. Consideration is given to this treaty as well as to reform of the International Health Regulations (IHR) as our principal governing instrument to prevent and mitigate future pandemics.

**Main body:**

Reasons exist to continue to work with the IHR as our principal governing instrument to prevent and mitigate future pandemics. All WHO member states are party to it. It gives the WHO the authority to oversee the collection of surveillance data and to issue recommendations on trade and travel advisories to control the spread of infectious diseases, among other things. However, the limitations of the IHR in addressing the deep prevention of future pandemics also must be recognized. These include a lack of a regulatory framework to prevent zoonotic spillovers. More advanced multi-sectoral measures are also needed. At the same time, a pandemic treaty would have potential benefits and drawbacks as well. It would be a means of addressing the gross inequity in global vaccine distribution and other gaps in the IHR, but it would also need more involvement at the negotiation table of countries in the Global South, significant funding, and likely many years to adopt.

**Conclusions:**

Reform of the IHR should be undertaken while engaging with WHO member states (and notably those from the Global South) in discussions on the possible benefits, drawbacks and scope of a new pandemic treaty. Both options are not mutually exclusive.

## Background

In the run-up to this year’s World Health Assembly (WHA), the World Health Organization (WHO) and numerous member states made a concerted effort to garner support for an international pandemic treaty. On 30 March 2021, 26 heads of states, the President of the European Council, and the WHO Director-General signed and issued a commentary calling for the international community to work together “towards a new international treaty for pandemic preparedness and response” [1]. By the time the WHA met less than 2 months later, a proposal was issued by the EU and 32 member states of the WHO. It called (and received support) for a special session of the WHA to be convened from 29 November to 1 December 2021 expressly “to consider developing a WHO convention, agreement or other international instrument on pandemic preparedness and response” [2]. Advocates see the treaty as a means to provide “universal and equitable access to safe, efficacious and affordable vaccines, medicines and diagnostics for this and future pandemics”, as well as to build international cooperation to improve alert systems and data-sharing [2].

Since these calls for a pandemic treaty began, arguments both in favour and against it have been advanced. We review these and others and propose that consideration should be given to such a new instrument while weighing the potential of strengthening the International Health Regulations (IHR) (2005).

## Main body

### The IHR: time for another revision?

As an international instrument, the IHR was created to prevent and respond to acute public health emergencies that have the potential to cross borders and threaten people worldwide, precisely like COVID-19. The IHR being the primary law governing our global responses to public health risks, COVID-19 quickly laid bare its weaknesses and failings [3]. However, before moving forward with the development of a pandemic treaty, we need to establish whether a governance vacuum exists [4].

While reasons exist to continue to work with the IHR as our principal governing instrument to prevent and mitigate future pandemics, its strengths and limitations in addressing the deep prevention of future pandemics must be recognized:

1. The IHR was approved unanimously by all 194 member states of the WHO; developing consensus could be a challenge for a new pandemic treaty that arose from a subset of member states. That being said, 61 countries express interest in a pandemic treaty; momentum could build as both the stakes of future pandemics, and interest in averting them, rise.

2. The IHR provides the WHO with the authority to collect surveillance data and to issue recommendations on trade and travel advisories to control the spread of infectious diseases. It imposes obligations on states to report on domestic laboratory capacity for testing infectious diseases [3]. Although the IHR adopts a One Health approach requiring, for instance, reporting on possible animal borne illnesses with the potential to cross borders, and it focuses on outbreak surveillance, containment, and response, it lacks a regulatory framework to prevent zoonotic spillovers in the first place. This reflects a governance gap, especially as containment may not be feasible. With other recent outbreaks, such as SARS and MERS, arising from zoonotic spillover from wildlife trade and agricultural intensification [5, 6], a pandemic treaty could foster a paradigm shift to prevent emergent zoonoses.

3. The IHR has a funded, longstanding secretariat which oversees health security measures, such as the self-assessment annual reporting tool where state parties report electronically to the WHA each year. This secretariat has proven itself to be supple and quick. It convened an 18-member COVID-19 Emergency Committee which has met eight times these past 12 months, issuing statements after each session. Members of this committee are diverse and include members from states which have indicated they may not support a pandemic treaty (e.g., China, USA, Brazil, and Russia).

4. Although the IHR was amended in 2005 following SARS – a disease that is assumed to have emerged from the wildlife trade – it is imperative that more advanced multi-sectoral measures be adopted. This requires greater collaboration between the WHO, the World Organization for Animal Health (OIE), the Food and Agriculture Organization, the United Nations Environment Programme, the Convention on International Trade in Endangered Species of Wild Fauna and Flora, and the Convention on Biodiversity to address zoonotic spillover. Measures to ensure production and equitable access to vaccines could further be developed. Determining whether this can be accomplished through IHR revisions, or whether a new treaty is required, should be the first order of business when the WHO convenes its pandemic treaty discussions later this year.

5. Advocates believe a pandemic treaty will provide stronger enforcement mechanisms [5]. Despite clear legal obligations outlined in the IHR, most State Parties do not comply with all requirements largely due to financing challenges [7]. Funding for WHO (and a number of other UN agencies) also remains insufficient. This begs the question of the adequacy of resources in these agencies -- whether for IHR revision and improved compliance, or for new treaty negotiations and a secretariat. Two initiatives launched in 2021 could address financing gaps concerning pandemics: the WHO Working Group on Sustainable Financing and the G20 High Level Independent Panel on Financing the Global Commons for Pandemic Preparedness and Response. Leveraging these emergent funding mechanisms will be crucial. If the proposed treaty is a ‘framework convention’, it will require considerable time to define obligations through a series of Committee of the Parties meetings, suggesting a lengthy process. At the same time, as member states address competing priorities as part of pandemic treaty discussions, this could strategically focus attention on the prevention of future pandemics and perhaps more strongly so than a focus on IHR revisions.

### Meeting the challenges through a pandemic treaty

Advocates of a pandemic treaty see it as a means of addressing the gross inequity in global vaccine distribution, with the Global South critically undersupplied. In October 2020, India and South Africa requested a temporary waiver on sections of the World Trade Organization (WTO) Agreement on Trade-Related Aspect of Intellectual Property Rights (TRIPS). The waiver would allow manufacturers to increase the supply of vaccines without risk of ensuing trade disputes. This would be an important first step to create a quick, low-cost, and effective increase in the supply of vaccines to the Global South (assuming there is parallel pressure to ensure technology transfer by patent-holding vaccine manufacturers). Yet there remain a small number of WTO member states that did not support the waiver [8]. Will these same countries be willing to negotiate with the pharmaceutical sector to address this pressing global concern so central to a treaty? Or will they align themselves with the pharmaceutical industry’s opposition to anything that would weaken its intellectual property rights (IPR) that remain protected under TRIPS and other trade and investment agreements?

Other concerns about a new pandemic treaty also exist:

1. Too many of the WHO member states most in need of the protections a pandemic treaty might provide are not yet ready to join the negotiating table, particularly those in the Global South [9, 10]. There may also be hesitancy among Global South member states to revise the IHR, although given the IHR is an existing agreement this may be less so. In either case, wide-spread representation from the Global South is imperative for the success of IHR revisions or a pandemic treaty. Enabling greater access to the vaccine through reform of IPR trade and investment rules could serve as an incentive.

2. The 2005 Framework Convention on Tobacco Control (FCTC) was the first instrument for which the WHO used its constitutional authority to develop international conventions to advance global health. It took 12 years to be adopted. While the COVID-19 pandemic incites greater urgency than did tobacco control, the complexities of vaccine manufacturing and inequities in its supply and distribution suggest the steep uphill battle a pandemic treaty will face. Patent-holding pharmaceutical corporations are certain to oppose any measures that could weaken their existing IPR protection in trade and investment treaties. Unlike provisions that excluded tobacco corporations from FCTC negotiations, it is unlikely that a pandemic treaty would seek similar exclusion of pharmaceutical companies from its negotiations. More optimistically, just as an international movement mobilized to ensure access to HIV-AIDS medicines in low- and middle-income countries eventually securing a TRIPS waiver [11], the proposed pandemic treaty could galvanize a similar global movement leading to new obligations that would go beyond those possible in IHR revisions.

3. At the same time, concerns exist that the current global reform momentum could mean lengthy pandemic treaty negotiations [12], whereas revisions to the IHR may be easier and more rapid to accomplish. That the post-SARS revisions to the IHR nonetheless failed to prevent or contain the spread of the COVID-19 pandemic at the very least argues for the necessity of a more robust governance framework with sufficient global and state level multi-sectoral cooperation and capacity. Whether a new treaty is better able to deliver on this than IHR revision remains the critical question, for which there is still no definitive answer.

## Conclusions

We are arguing in this Commentary neither for or against a pandemic treaty so much as proposing that we should first ensure that such a treaty will add substantially to pandemic prevention and intervention measures (such as equity in vaccine access) that are unlikely to occur through revisions to the IHR. Knowing what we know from our experience with COVID-19, it is clear the IHR is inadequate and considerable strengthening is needed. Vinuales et al. [5], for example, suggest an opportunity for stronger global One Health regulations. Although the concept of a new treaty has the potential to improve prevention and preparedness if structured effectively, delays remain a concern. A paradigm shift in global pandemic governance is needed, whether through IHR reform or a new pandemic treaty. At this juncture in planning for better pandemic preparedness and intervention, these two options should not be seen as mutually exclusive.

## Data Availability

Not applicable.

## Abbreviations

COVID-19: Corona Virus Disease-2019
FCTC: Framework Convention on Tobacco Control
IHR: International Health Regulations
IPR: Intellectual property rights
MERS: Middle East Respiratory Syndrome
OIE: World Organization for Animal Health
SARS: Severe Acute Respiratory Syndrome
TRIPS: Agreement on Trade-Related Aspect of Intellectual Property Rights
WHA: World Health Assembly
WHO: World Health Organization
WTO: World Trade Organization
